# The use of a task through virtual reality in cerebral palsy using two different interaction devices (concrete and abstract) – a cross-sectional randomized study

**DOI:** 10.1186/s12984-020-00689-z

**Published:** 2020-04-29

**Authors:** Andréa Fernanda Leal, Talita Dias da Silva, Priscila Bianchi Lopes, Shayan Bahadori, Luciano Vieira de Araújo, Murillo Vinicius Brandão da Costa, Íbis Ariana Peña de Moraes, Ricardo Henrique Marques, Tania Brusque Crocetta, Luiz Carlos de Abreu, Carlos Bandeira de Mello Monteiro

**Affiliations:** 1 Laboratório de Desenho e Escrita Científica, Departamento de Ciências Básicas, Faculdade de Medicina do ABC, Santo André, SP Brazil; 2 Departamento de Cardiologia, Escola Paulista de Medicina, Universidade Federal de São Paulo, São Paulo, SP Brazil; 3 Grupo de Pesquisa e Aplicações Tecnológicas em Reabilitação, Escola de Artes, Ciências e Humanidades - EACH - Universidade de São Paulo, São Paulo, SP Brazil; 4 Faculdade de Medicina, Universidade Cidade de São Paulo – UNICID, São Paulo, SP Brazil; 5 Programa de Pós-Graduação em Ciências da Reabilitação, Faculdade de Medicina da Universidade de São Paulo, Rua Cipotânea, 15, São Paulo, SP CEP: 05360-160 Brazil; 6 Orthopaedic Research Institute, Bournemouth University, Executive Business Centre, Holdenhurst Road, Bournemouth, BH8 8EB UK; 7 Programa de Pós-Graduação em Bioengenharia, Universidade Brasil, São Paulo, SP Brazil; 8 Secretaria de Tecnologia da Informação e Comunicação, Universidade do Estado de Santa Catarina – UDESC, Florianópolis, SC Brazil

**Keywords:** Cerebral palsy, Virtual reality exposure therapy, Learning, Motor activity, Motor skills

## Abstract

**Background:**

Cerebral Palsy (CP) is characterised by variable difficulties in muscular action, resulting in inability of the individual to perform functional movement. An option to provide functionality to the individual with CP is the use of computer innovation. The aim of this paper was to verify if there was any performance improvement in a task performed in a virtual environment and if there was transfer to the task performed in the real environment and vice versa in this population.

**Methods:**

A computer program was developed comprising a motor task, but with two possibilities of user interaction: a) concrete interface (with physical contact): in which the individual touches the computer screen to finish the task and b) abstract interface (no physical contact): in which the individual performs a hand movement in front of the Kinect device. Participants were split into two groups. The experimental group consisted of 28 individuals with CP within the ages of 6 and 15 years old. The control group included 28 typically developing individuals mirroring the age and sex of the experimental group.

**Results:**

Individuals from both groups were able to improve task performance and retain acquired information. The CP group presented worse performance than the control group in all phases of the study. Further findings showed that the CP group presented better performance in the abstract interface than in the concrete interface, whereas, in the control group, the opposite occurred: their best performance was in the concrete.

**Conclusions:**

Motor tasks performed by individuals with CP through an interface with a more virtual environment feature (abstract interface: Kinect) provided better performance when compared to an interface with a more real characteristic (concrete interface: Touchscreen).

**Trial registration:**

ClinicalTrials.gov Identifier - NCT03352440; Date of registration - November 17, 2017.

## Introduction

Cerebral Palsy (CP) is defined as a group of permanent disorders of the development of posture and movement, causing limitation in activities, which are attributed to a non-progressive disorder that occurs in foetal brain development or in infancy. Motor disorders in CP are often accompanied by disturbances of sensation, perception, cognition, communication, behaviour, epilepsy and secondary musculoskeletal problems [1]. Additionally, individuals with CP present complex motor skill disorders resulting in an abnormal muscle tone, which has a negative impact on posture, balance, motor coordination, muscle weakness, contractures and bone deformity [2]. Consequently, many children with CP experience difficulties in acquiring novel motor skills, which may lead to poor performance in activities of daily living and restricted participation [3–5].

Due to the severity of disability of individuals with CP, the rehabilitation path can be long and arduous. Thus, with the advancement of technology and greater accessibility to electronic devices, a growing and successful integration of Virtual Reality (VR) into the field of rehabilitation has been observed [6–8]. According to Kourtesis et al. [9], VR presents some adverse symptomatology (specially in immersive VR) such as nausea, dizziness, disorientation, fatigue, and instability. Also, other studies suggested further limitations, for example, frustration for the failure of the interface to detect movement or actions [10–12] and difficulty with hand-held interfaces mainly in positioning users with movement and postural impairments [13, 14]. Nonetheless, it should be considered that different studies presented positive aspects such as improvement in balance and mobility in adolescents with CP, increasing their level of independence in daily life activities [15, 16]. Moreover, the advantages of virtual reality include practice at home independently (i.e., online) or in interaction with others (e.g., e-games) and with or without supervision of a professional [3]. VR based-therapy allows patients to engage in a rehabilitation program with motivation and enjoyment [17, 18], increasing the adherence [19] and this characteristics can optimise motor learning, which can later lead to neuroplastic changes [18] and those advantages can be the target for future clinical approach for individuals with CP.

Despite the positive use of VR in the treatment of individuals with CP, studies have noted that the possibility to transfer performance improvement from a VR task to a real task should be carefully considered, and as such further research is required [3, 17]. According to Monteiro et al. [3], in virtual environments (VE), participants may execute a determined task with a relatively abstract aim directed at intangible objects, and this can directly influence performance. Executing a task in a VE requires a spatial-temporal organisation, which differs from the real environment (RE), especially among people with movement disorder. Thus, a task that involves a direct interaction with the environment, including physical contact, generates a richer pool of tactile and proprioceptive information that can be used to adapt and guide movement, than a more abstract task in a virtual environment [3]. However, real tasks generally require fine control of functions, and hence, a greater accuracy of movement to touch an object. Due to alteration in muscle coordination and co-contraction, individuals with CP present more difficulty in performing such functions accurately [20].

On the other hand, virtual tasks using technology such as a webcam or Kinect system do not require accurate coordination, nor do they require use of fine movements (finger-function). Thus, the possibility to use proximal patterns of movement (arm and shoulder movement) in the virtual device can provide better function for individuals with CP [21].

In other words, the differences between real and virtual environments could be explained based on the principle that the movement information is specific to tasks and situations. The ecological approach of perception and action [22] supports that, to each action, a specific set of information-movement is built. This difference might be evidenced when comparing similar actions using different interaction devices; i.e. with physical interaction (e.g. with touchscreen or tactile feedback) or Abstract interaction (without touch using Kinect or webcam). These findings are also aligned to the neuropsychological statements, which define that the action of catching a real object involves specific parts of the tactile and visual system (i.e. because the virtual experience is not exactly the same as the real, different areas of the sensory-motor system are activated) [23, 24].

For instance, Van Der Weel et al. [25], and Monteiro et al. [3] compared the performance of people with posture and movement alterations in two tasks of interception, which differed in their degree of abstractness (i.e. more tangible with physical contact or abstract, in this case using a hand movement – waving gesture). Results have shown performance differences between real and virtual environments in people with posture and movement alterations. Although, the tasks were similar, differing only in the interaction devices, the performance in the RE was better than that observed in the VE. However, a more recent study from Martins et al. [21], that used touchscreen (representing concrete task) and a webcam (representing abstract task) during a coincident timing task, showed that individuals with CP did not required a big effort to control their movement to reach the target when performing virtual tasks. Thus, improvement of technology (i.e. with modern, devices with more abstract characteristic), enhances the possibility for individuals with CP to perform a motor task with less coordination, acceleration, and deceleration of movement than is needed in concrete tasks [20, 21].

As mentioned before, this study selected a 3D computer game that could be executed with a concrete (Touchscreen) or abstract interface (Kinect system) in individuals with CP. Therefore, the aim of the present study was to investigate if there was an improvement in performance of such population during an abstract task, and if there was any motor transfer to the following concrete task. Thus, individuals with CP and typically developing (TD) individuals practiced a task differed in the device interaction, considering the degree of abstractness. So, during the concrete task the participants were asked to complete the target using a touchscreen interaction, using their fingers to touch and burst the object. During the abstract task, the participants were asked to interact using a Kinect system device through execution of a hand movement, i.e. waving gesture.

The hypothesis is that individuals with CP will present worse performance during all the study phases, compared to typical individuals. However, considering the motor difficulty that characterises CP, even with individuals’ unfamiliarity with the technology or task, it is hoped that the wave gesture using an abstract task will provide better performance for those in the CP group. If the hypothesis is verified, it will reinforce the importance of identifying functional characteristics for the use of VR in clinical practice, to improve performance of individuals with CP.

## Methods

### Participants

**Fig. 1:**
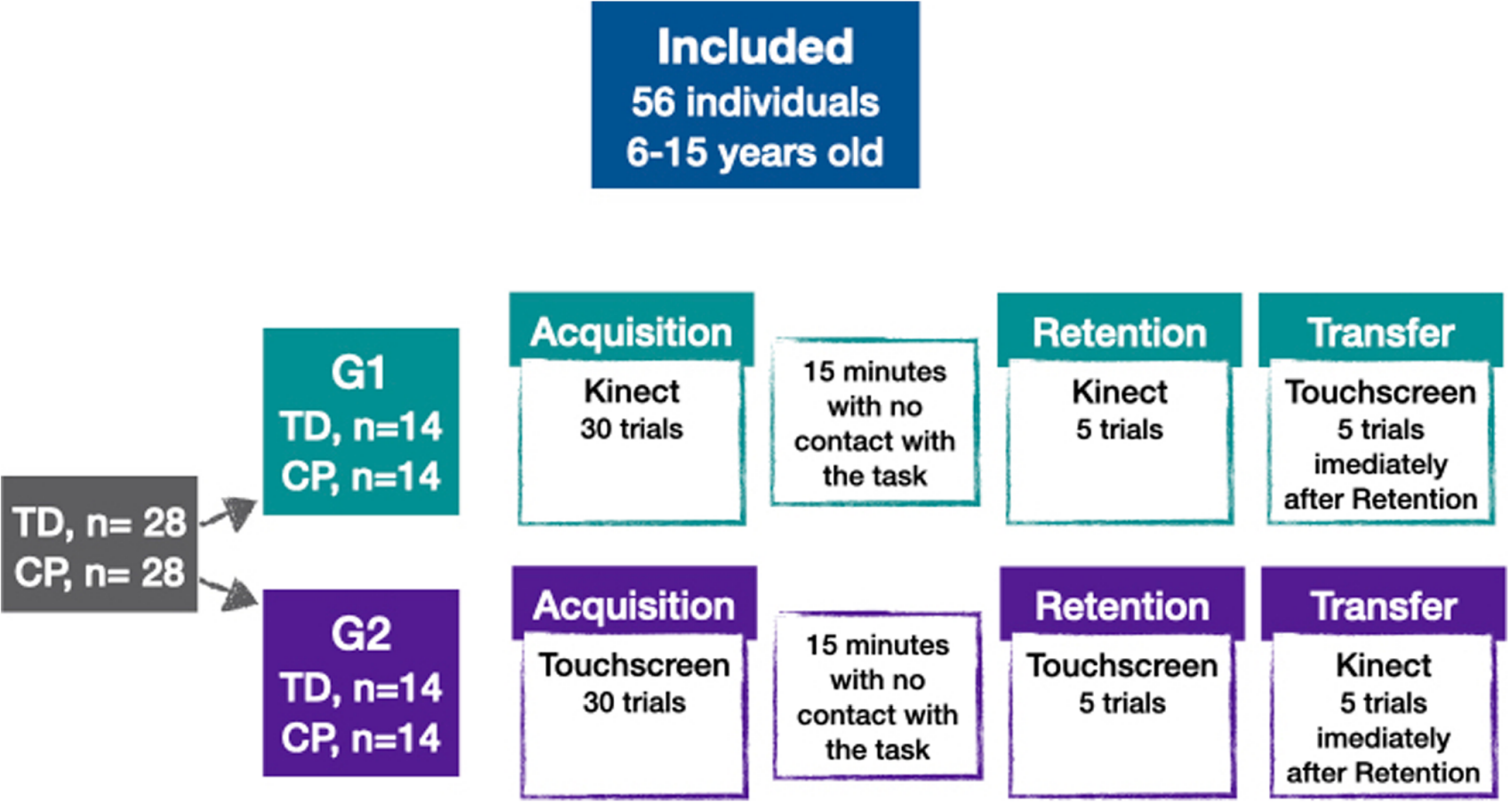
Design of the study. TD: Typical Development; CP: Cerebral Palsy; G1: Performed practice of the task using an abstract interface; G2: Performed practice of the task using an concrete interface

A total of 56 individuals were included in this study, 28 individuals with CP who constituted the experimental group and 28 TD individuals who constituted the control group. All were aged between 6 and 15 years (detailed info about participants are presented as Supplementary Material – Table S1). Participants were further divided into four subgroups. Two subgroups practiced the task using an abstract interface (Kinect): 14 formed the experimental group 1 (CP-Group1) and 14 individuals formed the control group 1 (TD-Group1). The remaining two subgroups practiced the task using a concrete interface (Touchscreen): 14 formed the experimental group 2 (CP-Group2) and 14 individuals formed the control group 2 (TD-Group2) (Fig. 1). All control groups were matched for age and sex with the experimental group.

Inclusion criteria: for the experimental group, individuals with CP, with diparesis and spastic hemiparesis, were included; they also were classified as levels I, II and III, according to the Gross Motor Function Classification System (GMFCS) [26, 27], and I, II and III according to the Manual Ability Classification System (MACS). Thus, all individuals with CP who were evaluated could stay seated, had function of upper limbs and had independent walking, even though this was with the aid of a walker or crutches.

Exclusion criteria: individuals who had undergone surgery or chemical neuromuscular blockade for less than 6 months in the upper limbs were excluded. Also excluded were those who had other diseases or changes in cognitive functions that preclude cooperation and understanding in the proposed activities. In this study, none of the subjects invited were excluded.

This study was approved by the Ethics Committee of ABC Medical School research (Santo André, Brazil), under protocol number CAAE 32476414.5.0000.0082. The participants and/or their legal guardians provided written informed consent.

### Material and apparatus

In this study, we used the Check Limit Game [28]. This game allows the use of a Kinect sensor for motion capture (abstract interface), as well as a Touchscreen (concrete interface). The VR tool used was developed by the Information Systems Laboratory of the School of Arts, Sciences and Humanities of the University of Sao Paulo (Escola de Artes, Ciências e Humanidades da Universidade de São Paulo - EACH/USP) which features on the computer a task in which 96 bubbles (distributed in rows and columns) are on the screen and must be touched using finger contact or sliding the finger to other bubbles (touchscreen group) or using an avatar hand (Kinect group) to change colour from green to light purple. The goal is to change the colour of a maximum number of bubbles in 15 s. Data was collected and performance was analysed based on the number of bubbles hit.

### Procedure and design

**Fig. 2:**
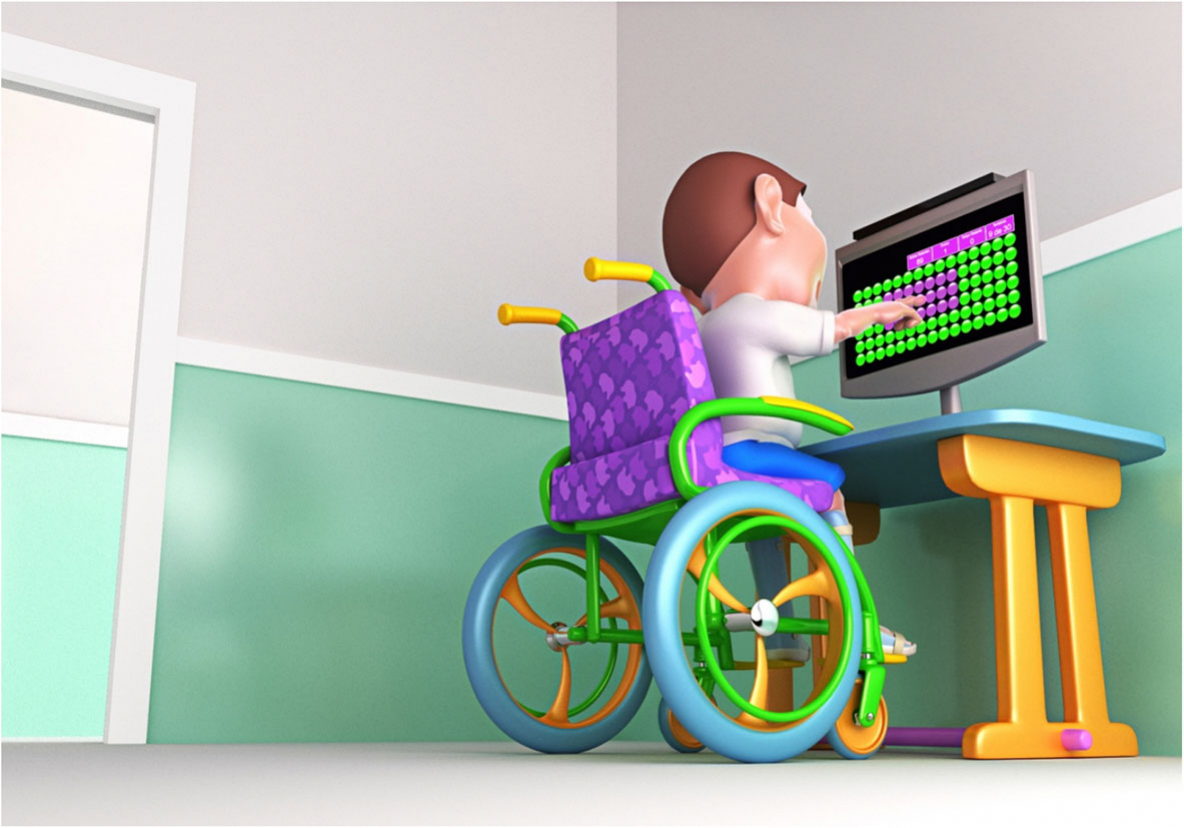
Representation of the child performing a motor task through the Touchscreen. Source: Own authorship

**Fig. 3:**
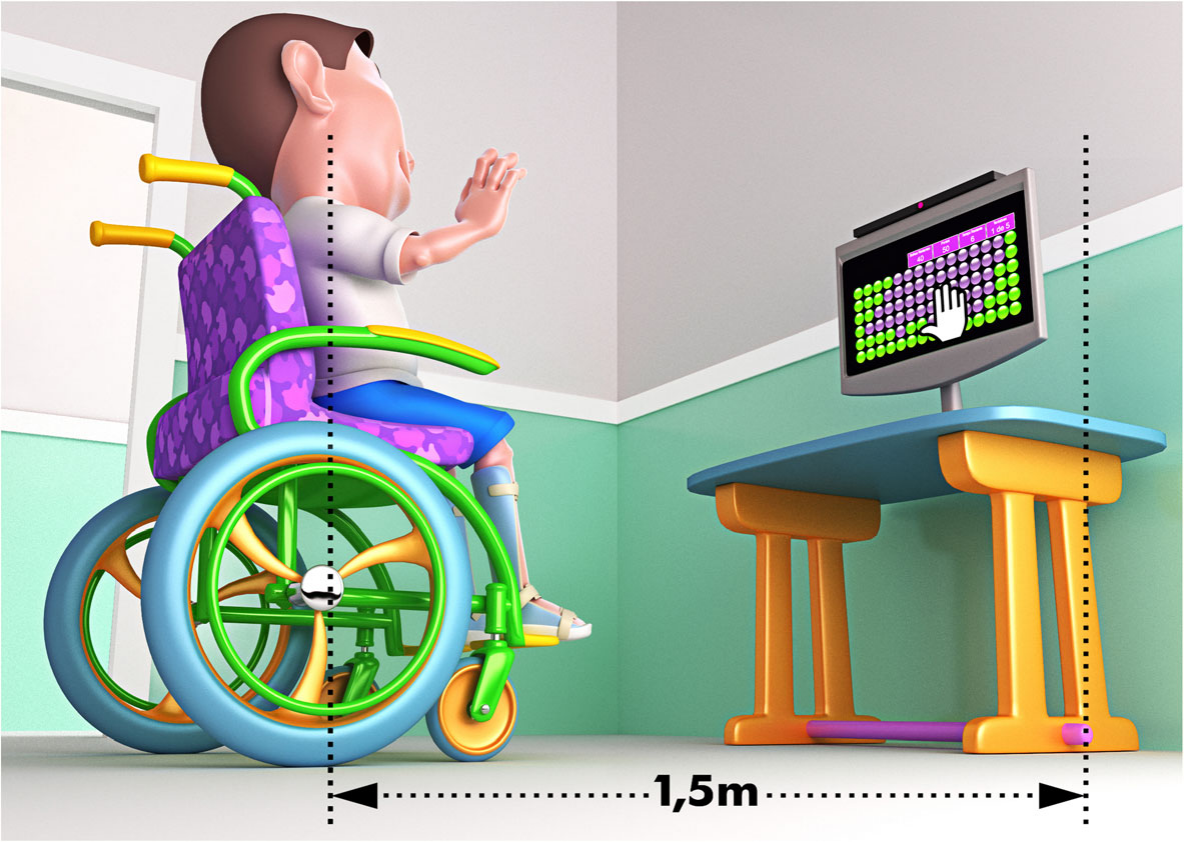
representation of the child performing a motor task through Kinect. Source: Own authorship

To perform the task, participants who performed the concrete interface (Touchscreen) were positioned in front of the computer so that they were adjusted in height (this depended on the physical composition of each participant) and at a comfortable distance to allow their hand to reach the computer screen. The participants who performed the abstract interface (Kinect) were positioned approximately 1.5 m away from the computer screen to provide the Kinect system movement capture (Figs. 2 and 3). To counterbalance across groups, participants of both the CP group and the TD group were randomly assigned to the subgroup (abstract or concrete interface).

Participants were instructed to place their preferred hand (e.g. the less impaired hand) on a mark in front of the computer or on the top of their thigh, depending on whether they were in the abstract interface group or the concrete interface group respectively. Once the bubbles appeared on the computer screen, the individual had 15 s to get as many bubbles as possible, moving the preferred hand to either touch the target (bubbles) on the screen or to make a hitting gesture in front of the Kinect system. During the task, the protocol was divided into three phases - Acquisition phase: with 30 trials (15 s each), the acquisition phase lasted 7 min and 30 s; after acquisition phase participants stayed 15 min of no contact with the task and started the Retention phase: with 5 trials performed with the same interface used in acquisition and lasted 1 min and 15 s; and Transfer phase: after retention participants executed 5 trials with change of interface to verify transfer and lasted 1 min and 15 s (Fig. 1).

### Data analysis

The dependent variable used was the number of bubbles reached. The dependent variable was submitted to a 2 × 2 × 2 (group: CP/TD, interfaces: abstract/concrete, blocks, respectively) ANOVA with repeated measures on the last factor. For the factor block, the results were obtained using blocks (average of five attempts each) for all study phases (acquisition phase had 30 attempts divided in 6 blocks - A1 to A6; retention had 5 attempts, so one block - R; and the same apply for the one transfer block - T). Thus, separate comparisons were made for acquisition (first acquisition block – A1 versus last acquisition block – A6), retention (A6 versus retention block - R) and transfer (R versus transfer block - T). Post hoc comparisons were carried out using the Tukey-LSD (Least Significant Difference) [29] test. Partial eta-squared (ŋ_p_^2^) was reported to measure effect size and was interpreted as small (effect size > 0.01), medium (effect size > 0.06), or large (effect size > 0.14) [30]. Findings were significant at *p* < 0.05.

## Results

Data about descriptive statistics is presented on Table 1.

**Table 1:** Descriptive statistics represented by Mean (Standard Deviation) of number of bubbles reached by blocks and Age. *p*-values represent comparison between abstract interface (K) and concrete interface (TS) subgroups regarding age and bubbles reached in each block of trials (A1-A6, R and T)

	CP Group			TD Group		
	K	TS	***p***	K	TS	***p***
**N**	14	14		14	14	
**Age**	11.9 (3.0)	10.5 (2.4)	.606	11.3 (3.0)	11.0 (3.5)	.885
**A1**	62.4 (12.9)	58.5 (21.9)	**.007**	84.5 (8.3)	86.1 (14.8)	.167
**A2**	70.1 (10.0)	60.9 (22.3)	**.002**	86.5 (6.3)	88.4 (12.1)	**.050**
**A3**	69.8 (8.1)	59.1 (20.2)	**.001**	86.3 (7.2)	90.8 (9.3)	.623
**A4**	68.8 (11.3)	59.3 (22.2)	**.010**	86.3 (8.3)	90.6 (8.6)	.659
**A5**	70.5 (13.5)	60.0 (23.3)	.063	88.6 (6.5)	91.1 (8.0)	.605
**A6**	71.5 (12.6)	62.3 (21.1)	**.046**	88.3 (7.0)	92.7 (7.1)	.736
**R**	72.2 (10.6)	66.1 (20.5)	**.013**	89.3 (7.6)	93.3 (4.8)	.215
**T**	60.9 (21.7)	68.8 (12.3)^a^	**.019**	88.3 (7.2)	77.5 (11.0)	.245

*a* Missing 1 case; *CP* Cerebral palsy; *TD* Typical development; A1 … A6: acquisition blocks; *R* Retention; *T* Transfer with opposite interface; *K* Kinect interface - Abstract; *TS* Touchscreen interface - concrete

All values of bubbles reached in the subsequent sections are represented as Mean (M).

### Acquisition

**Fig. 4:**
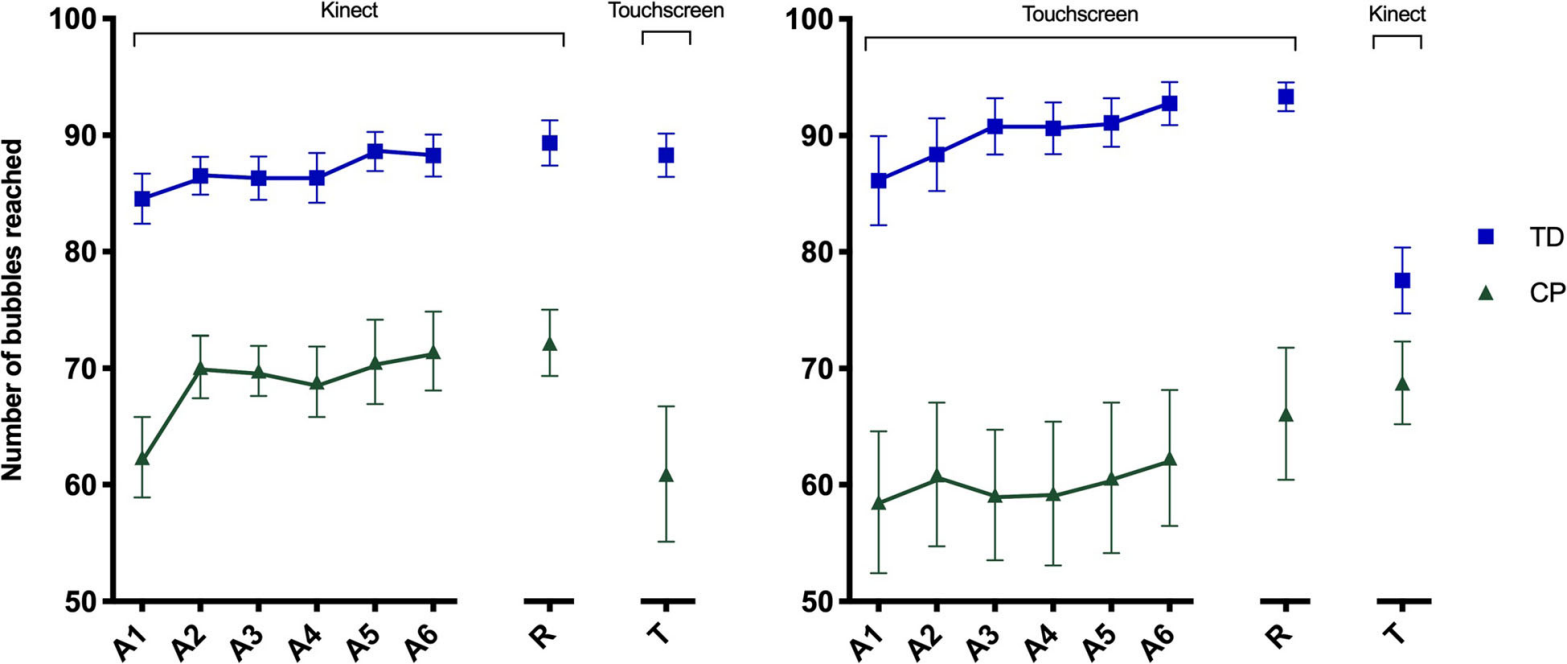
Representation of the blocks of trials in groups

The number of bubbles reached is illustrated in Fig. 4. Significant effects were found in Blocks, F (1, 53) = 19.2, *p* < .001, ŋ_p_^2^ = .27, and Group, F (1, 53) = 48.8, *p* < .001, ŋ_p_^2^ = .48. This result suggests that participants have increased the number of bubbles reached from the first Block (A1) (M = 72.8) to the last Block (A6) (M = 77.7) and the CP-group had worse performance (M = 63.7) when compared to the TD-group (M = 87.9). Post hoc comparisons showed that in the CP-group, the performance between Blocks A1 and A6, was better in abstract interface (M = 62.4 and M = 71.5, respectively), and there was no difference in concrete interface. In the TD-group, the opposite occurred: there was a significant increase in the number of bubbles reached in concrete interface from Block A1 (M = 86.1) to Block A6 (M = 92.7), but this did not occur for abstract interface.

A1 – A6: blocks of acquisition phase; R = block of retention test; T = block of transfer with opposite interface; CP: group with Cerebral Palsy; TD: group with Typical Development.

### Retention

Marginal significant effects were found for Blocks, F (1, 53) = 3.5, *p* = .066, ŋ_p_^2^ = .06, and significant effects for Group, F (1, 53) = 51.2, *p* < .001, ŋ_p_^2^ = .49. This result shows that the participants increased the number of bubbles reached from the last Block of attempts (A6) (M = 78.7) to the Retention (R) (M = 80.2) and the CP-group had worse performance (M = 68.0), when compared to TD-group (M = 90.9).

### Transfer with opposite interface

Significant effects were found for Block, F (1, 52) = 17.7, *p* < .01, ŋ_p_^2^ = .25 and Group, F (1, 52) = 41.6, *p* < .001, ŋ_p_^2^ = .45. This result suggests that the number of bubbles that participants reached decreased from R (M = 80.8) to T (M = 73.9). Additionally, the TD-group reached a higher number of bubbles (M = 87.1) than the CP-group (M = 67.5).

Interactions between Block by Group by Interface, F (1, 52) = 16.5, *p* < .001, ŋ_p_^2^ = .24 were found. The post hoc test showed that in the Abstract interface, for the CP-group there was a significant decrease from block R (M = 72.2) when transferring to the Concrete interface in block T (M = 60.9). For the concrete interface, when transferring to abstract interface, this difference was not significant (M = 68.2 to 68.8, respectively). However, for the TD-group a significant decrease was observed from retention on concrete interface (M = 93.3) to transfer on abstract interface (M = 77.5), while for retention on abstract interface with transfer to concrete interface there was no significant difference (M = 89.3 to 88.3, respectively).

## Discussion

The current study aimed to investigate whether the performance of individuals with CP and typically developed individuals improved during a task practice in an abstract interface (Kinect) compared to a concrete interface (touchscreen) and if transfers of performance existed between these two environments.

The results have shown that the group of individuals with CP, as hypothesised, presented worse performance in all phases of the study, i.e. a lower number of reached bubbles than the control group. This difference in performance between individuals with CP and individuals with TD was also noted in previous studies, such as: Hung & Gordon [31] in bimanual tasks; Burtner et al. [32] in coordination movements of upper limbs and Monteiro et al. [3] who performed tasks of coincident timing on a computer with and without physical contact. Thus, it is established that individuals with CP explore both abstract and concrete environments in different manners, and with more difficulty, compared to people with typical development [25, 33]. This difference between groups could be justified by sensory and motor alterations in individuals with CP that result in greater difficulty in movement [34, 35].

Considering the results of the present study, when comparing interfaces (touchscreen/concrete and Kinect/abstract), it can be observed that the acquisition phase was marked by significant effects for the CP and TD group, but with opposite results comparing interfaces: the CP group has presented a higher increase in performance (i.e. reached bubbles from A1 o A6) in the abstract interface compared with concrete, whereas the opposite occurred in the TD group, and as such there was a significant increase in concrete interface performance. Also, regardless of the phase of the study (acquisition, retention or transfer) when we run separate analysis between abstract and concrete interfaces on each group in all blocks of attempts (Table 1), we’ve found similar results than in improvement in performance, i.e. that the CP group showed significant higher performance in the abstract interface comparing to concrete interface. However, for the TD group, the higher performance on concrete interface was not significant.

These findings are perhaps the most important result of the present research and could significantly contribute towards rehabilitation research. This is because they have indicated that an abstract task (using Kinect system) provides improvement and more important better performance regarding number of bubbles reached for individuals with CP as compared to a concrete task (Touchscreen).

As hypothesized, such findings can be explained by the fact that the use of abstract device does not require perfect motor coordination, nor does it require the use of fine movements (finger function), which are impaired in most individuals with CP. Thus, the possibility of using proximal patterns of movement (arm and shoulder movement) in the abstract device, without the necessity for finger movement, has provided better function for individuals with CP. This is consistent with Martins et al. [21] who state that devices with no contact required more global movements with the participation of more proximal movement patterns. Thus, participants with CP were able to adapt more to the virtual task and presented better performance. However, touchscreen tasks imply precise control of functions and, consequently, require a greater accuracy of movement [29, 36].

During the concrete task, the individuals with CP would have had to significantly control their preferred hand to touch the computer screen, which requires accuracy with more adequate acceleration and deceleration of movement. As cited by Fernani et al. [20], individuals with CP presented greater difficulty when performing tasks that require more precision of movement rather than velocity. Thus, based on our results, the concrete task requires more precision and fine movement, while abstract tasks have the characteristic of speed with global movements. It is also known that individuals with CP have proprioceptive and tactile alterations [35, 37]. Therefore, it can be speculated that as the abstract task proposed does not require physical contact, this characteristic of the Kinect system provided a better adaptation to the task, leading to better performance in reaching the bubbles for individuals with CP.

Comparing the abstract and concrete tasks, both depend on a different connection of information (see Monteiro et al. [3]. Additionally, a task involving direct interaction with the environment, including physical contact, perhaps generates a richer set of information to guide the movement than a more Abstract task in a VE. Thus, the non-impaired nervous system that characterises the TD group had benefits from this tactile task, and can justify the better performance for TD group only during the practice of the concrete task.

To verify whether there was motor learning or not, the observation of performance during the acquisition phase is not enough. Retention and transference tests are widely used to find out the inference of motor learning in people without disabilities [38, 39], those with motor disabilities [29, 36, 40] and even in studies with CP [3, 41]. In the present research the retention test showed that participants of both groups retained the acquired performance, on both interfaces, i.e., the practice of virtual tasks, in an environment with touch as well as without it, promotes short-term retention, which indicates the ability to keep the acquired information. However, relating to the transfer phase, it could be verified that the individuals with CP that have improved performance on acquisition in abstract task were not able to transfer it to the concrete. Regarding the control group, the opposite result could be observed, i.e. individuals with TD have presented better performance in concrete task, although they were unable to transfer it to the abstract.

These results are partially in agreement with Monteiro et al. [3] who investigated the training effect, comparing virtual and real environments, through simple tasks of coincident timing in the computer. Different from our results they found that individuals with CP were able to improve their performance in both environments, although as we found there was no transference from the virtual to the real environment.

Moreover, some interesting speculation and proposals for future studies can support these results:

### Practice during acquisition

The practice of a difficult task during acquisition phase likely promoted better transfer for an easier task thereafter – it could be seen when considering the abstract device (Kinect) interaction for the TD group and the concrete device (Touchscreen) for the CP group, that lead to the worst performance during acquisition, but allowed for better transference of performance for both groups shortly afterwards. Following this thought, in the study of Martins et al. [21] with individuals with CP, a virtual coincident timing task practice with upper limb movements in front of a computer webcam was used, with the aim of virtually intercepting spheres that fell in four rows following the rhythm of a pre-selected song. The authors showed that this practice in VE was more difficult than in the RE one, but provided better performance in a more real task (using the touchscreen) thereafter.

Although the speculation that the abstract task for TD group and concrete task for CP group presented much more difficultly during practice and could be a reason for the effectiveness of transfer, this is a factor that should be scrutinised in the future. Studies using a protocol with a more difficult abstract task during practice for individuals with CP will possible provide better performance in the transfer phase for a concrete environment and if it is proved, can be an interest result for an effective use of VE during rehabilitation program.

### Technology

Improvement in technology will promote better interaction and environment control. The sensitivity of the motion device is improving, we can hypothesized that Kinect system sensibility used in this study to collect data in 2017 was higher than, for example the available webcam in the period of data collection of the Monteiro et al. [3]. Those authors investigated in 2013 the training effect, comparing virtual and real environments, through simple tasks of coincident timing in the computer using a webcam. Different from our results they found that individuals with CP were able to improve their performance in both environments, although as we found there was no transference from the virtual to the real environment. Perhaps, the new technology used in our study (Kinect system), could have contributed towards a better result using the virtual device for individuals with CP. With the rapid advances in technology in this moment, even Kinect system can be considered an old device, there are newer webcams that are able to provide better movement capture and subsequently better improvements in performance for individuals with CP (see Martins et al. [21]). With an enhanced accuracy in sensory devices and computer, it may be possible to create a variety of tasks focused on rehabilitation. In a similar way than Martins et al. [21], these tasks can offer a range of variables to be used for individuals with CP. According to Crocetta et al. [28], the improvement of technology will allow the development of VR rehabilitation software package with possibilities to adapt the task to the objective of the rehabilitation program, in addition to the space and time constraints of the therapy. Moreover, such software will allow the therapist to organise their program with the possibility to control variables and parameters in order to obtain the participant necessary sequence execution of movements performed. The movement may include: positions reached, directions of movement, response time execution, memory and overall temporal accuracy. Those possibilities will be the future for clinical practice.

For instance, treatment programs tailored to individuals with CP make use of VE in order to improve patients’ functionality. Although these programs are successful regarding adherence [19], it has still not been consistently proven if there is an improvement in more VE tasks, how it can help functionality for individuals with CP and whether there is transference-improving performance in REs. Regardless our positive results using the virtual device, we agree with Monteiro et al. [3] in that careful implementation is required when attempting to enhance motor functioning of individuals with CP in their daily natural environment. It is likely that interfaces without physical contact can promote independence and functionality in routine tasks for individuals with CP, but careful consideration is required with regards to using VR as an intervention with the propose of providing transfer to a real task. Further investigation is therefore required to support the use of VR in cerebral palsy treatment.

### Limitations

Although we found interesting results, we can point out some limitations of the present study: [1] we did not assess the engagement and satisfaction with those two devices used. Meyns et al. [42] presented that the use of virtual reality games as a rehabilitation strategy has been growing because it increases the child’s participation in a motivational and engaging way, subsequently, improving performance and functional results. Thus, as in our research protocol, the virtual task promoted better function, and we can speculate that the motor strategy and interaction promoted by a virtual task (e.g. engaging game) has contributed to this performance. However, we did not analyse this factor, and this can be considered as an area for further research [2]. We compared two tasks, abstract and concrete, but with different pattern of movement (differing accuracy demands and levels of difficulty), it could influence the results and the discussion. We agree with Moraes et al. [43] that the pattern of movement analysis in a virtual task could provide some interesting results for discussion and is important to be considered for further studies. Perhaps using a virtual task and comparing with a real task (with a real object) and preserve the pattern of movement between both tasks could be the next step to be investigated [3]. Lastly, it is also important to emphasize that the participants could have reached a potential “ceiling effect”, as found by Moraes et al. [43], following the second or third block of the practice and it could have influenced the results.

## Conclusion

The motor task performed through an abstract interface (no physical contact - Kinect) in children with CP resulted in improvement in motor performance when compared to a concrete interface (with physical contact - Touchscreen). It was also found that there was retention of the task in the short term by children with Cerebral Palsy. Regarding transference of motor skill, the virtual environment did not promote transference to a real environment, probably because the real environment was more difficult (required more accuracy of movement). It is important to emphasise that virtual environments can be considered a functional tool to promote improvement and mantainance of motor performance for children with CP. Despite those positive findings we suggest a more rigorous design study with multiple levels of task difficulty using this platform (Bridge Games) to support and make more reliable statement.

## Supplementary information

**Additional file 1 Table S1.** Characteristics of subjects with Cerebral Palsy regarding age, gender, Gross Motor Function Classification System (GMFCS), Manual Ability Classification System (MACS) and type of Cerebral Palsy (type of CP).

## Data Availability

Supporting datasets can be obtained from Talita Dias da Silva: write at ft.taliatdias@gmail.com

## Abbreviations

CP: Cerebral palsy
VR: Virtual reality
ICF: International classification of functioning, disability and health
VE: Virtual environments
RE: Real environment
TD: Typically developing
GMFCS: Gross motor function classification system
MACS: Manual ability classification system
A1: First block (of mean of five attempts) of the acquisition phase
A6: Last block (of mean of five attempts) of the acquisition phase
R: Block (of mean of five attempts) of the retention phase
T: Block (of mean of five attempts) of the transfer phase
