# Supplementary material for: The use of a task through virtual reality in cerebral palsy using two different interaction devices (concrete and abstract) – a cross-sectional randomized study
Source: J Neuroeng Rehabil. 2020 Apr 29;17:59. doi: 10.1186/s12984-020-00689-z (PMC7191706; doi:10.1186/s12984-020-00689-z)
Supplement: Supplementary file 1 — Additional file 1 Table S1. Characteristics of subjects with Cerebral Palsy regarding age, gender, Gross Motor Function Classification System (GMFCS), Manual Ability Classification System (MACS) and type of Cerebral Palsy (type of CP). [file 12984_2020_689_MOESM1_ESM.docx]

**Table S1:** Characteristics of subjects with Cerebral Palsy regarding age, gender, Gross Motor Function Classification System (GMFCS), Manual Ability Classification System (MACS) and type of Cerebral Palsy (type of CP).

| Subject | Interface | Age | Gender | GMFCS | MACS | type of CP |
| --- | --- | --- | --- | --- | --- | --- |
| 1 | Kinect | 15 | Male | II | II | diparetic |
| 2 |  | 8 | Male | II | III | diparetic |
| 3 |  | 14 | Male | II | II | diparetic |
| 4 |  | 12 | Male | III | III | diparetic |
| 5 |  | 11 | Male | III | III | diparetic |
| 6 |  | 11 | Male | II | II | diparetic |
| 7 |  | 11 | Male | II | II | diparetic |
| 8 |  | 11 | Male | II | II | diparetic |
| 9 |  | 10 | Female | I | II | hemiparetic |
| 10 |  | 13 | Female | II | II | diparetic |
| 11 |  | 13 | Male | II | II | diparetic |
| 12 |  | 12 | Male | II | II | diparetic |
| 13 |  | 9 | Male | II | II | diparetic |
| 14 |  | 10 | Female | II | II | diparetic |
| 15 | Touchscreen | 10 | Male | II | II | diparetic |
| 16 |  | 13 | Male | III | III | diparetic |
| 17 |  | 11 | Male | II | II | diparetic |
| 18 |  | 13 | Male | II | II | diparetic |
| 19 |  | 10 | Male | II | II | diparetic |
| 20 |  | 12 | Male | II | II | diparetic |
| 21 |  | 9 | Male | II | II | diparetic |
| 22 |  | 12 | Male | II | II | diparetic |
| 23 |  | 9 | Male | II | II | diparetic |
| 24 |  | 7 | Female | II | II | diparetic |
| 25 |  | 15 | Female | II | II | diparetic |
| 26 |  | 8 | Male | II | II | diparetic |
| 27 |  | 8 | Female | I | I | hemiparetic |
| 28 |  | 15 | Female | II | II | diparetic |
